# Luminescent Nanocucurbits Enable Spatiotemporal Co‐Delivery of Hydrophilic and Hydrophobic Chemotherapeutic Agents

**DOI:** 10.1002/advs.202509782

**Published:** 2025-09-23

**Authors:** Ping Wei, Yunshan Ding, Shangning Liu, Jinhui Jiang, Jinghua Chen

**Affiliations:** ^1^ Key Laboratory of Carbohydrate Chemistry and Biotechnology Ministry of Education School of Life Sciences and Health Engineering Jiangnan University Wuxi 214122 China; ^2^ Department of Polymeric Materials School of Materials Science and Engineering Tongji University Shanghai 201804 China

**Keywords:** aggregation‐induced emission, drug delivery, nanocucurbits, self‐assembly

## Abstract

The generation of nanostructures with asymmetric morphologies has garnered significant attention in nanomedicine research, particularly since morphological characteristics have been shown to critically influence the biological functionality. Herein, the rational design and successful fabrication of luminescent cucurbit‐shaped nanoparticles (nanocucurbits) is presented for co‐delivery of hydrophilic and hydrophobic chemotherapeutic agents. These innovative nanostructures are obtained through the self‐assembly of a tetraphenylethylene (TPE)‐graft polypeptide copolymer, poly(ethylene glycol)_45_‐*block*‐poly[(L‐glutamic acid‐TPE)_26_‐*stat*‐(L‐glutamic acid)_29_] [PEG_45_‐*b*‐P(GATPE_26_‐*stat*‐GA_29_)], employing an optimized solvent‐switch method. Remarkably, these nanocucurbits exhibit a dramatically enhanced uptake level of which the total amount of intracellular endocytosis is about two‐fold higher than that of the spherical counterparts. Capitalizing on this distinctive asymmetric structure, the nanocucurbits exhibit an exceptional dual‐loading capability, enabling the simultaneous encapsulation of both hydrophilic (doxorubicin hydrochloride, DOX) and hydrophobic (camptothecin, CPT) agents. The co‐delivery of dual‐drugs within the same carrier, along with the sequential release of the drug combination, enables this drug delivery system with synergistic chemotherapeutic effect against hepatoma cells. Overall, the unique combination of morphological advantages, intrinsic luminescence, and dual‐drug loading capability renders these nanocucurbit‐based system a promising platform for multi‐agent combination therapy, particularly in cancer treatment requiring the simultaneous delivery of drugs with distinct physicochemical properties.

## Graphical abstract

A cucurbit‐shaped luminescent nanoparticle (nanocucurbit) is engineered through the self‐assembly of polypeptides conjugated with aggregation‐induced emission (AIE) fluorophore. The resulting nanocucurbit exhibits approximately two‐fold higher cellular internalization efficiency compared to its spherical counterparts. Capitalizing on their compartmentalized nanostructure, spatiotemporal co‐delivery of hydrophobic and hydrophilic chemotherapeutic agents can be achieved.

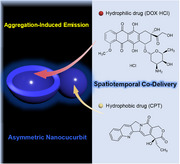

## 1 Introduction

Chemotherapy has emerged as one of the most effective treatment approaches in clinical oncology practice.^[^ ^1^, ^2^ ^]^ However, the inherent complexity and heterogeneity of tumors enable cancer cells to develop multiple mechanisms to evade chemotherapy‐induced cell death.^[^ ^3^ ^]^ These biological challenges severely limit the therapeutic efficacy of a single chemotherapeutic drug. Consequently, co‐delivery of different agents with various effects is becoming increasing crucial for eliciting diverse cytotoxic effects on cancer cells.^[^ ^4^, ^5^, ^6^ ^]^ Recently, nano‐biotechnology has found the way to partially address this issue by various nanomaterial‐based drug formulation techniques. A variety of delivery systems involving the co‐delivery of anticancer drugs have been developed, including liposomes, polymeric emulsions, and inorganic nanoparticles, aiming to achieve a synergistic antitumor effects.^[^ ^7^, ^8^, ^9^ ^]^ Nevertheless, interference between therapeutic agents and toxicity concerns associated with multiple‐component carriers are inevitable in these co‐delivery systems.^[^ ^10^, ^11^ ^]^ Comparatively, nanocarriers designed based on biodegradable and environmentally friendly components demonstrated unique advantages in co‐delivery of therapeutic drugs, thereby avoiding aforementioned issues.

Owing to their inherent biocompatibility, biodegradability and bioactivity, polypeptides are ideal building blocks for engineering functional nanomaterials through spontaneous organization, which hold significant potential in reducing toxicity, improving drug targeting, and enhancing drug delivery efficiency.^[^ ^12^, ^13^ ^]^ Given the wide variety of amino acids (including both natural and non‐natural ones), peptides can be designed to exhibit diverse chemical and physical functions.^[^ ^14^, ^15^, ^16^ ^]^ Critically, polypeptide chains facilitate multiple noncovalent interactions, such as hydrophobic interactions, hydrogen bonding, van der Waals forces, and π–π stacking interactions.^[^ ^17^, ^18^, ^19^ ^]^ The combination of several noncovalent forces together can generate relatively stable and well‐organized nanostructures, such as nanospheres,^[^ ^20^ ^]^ nanofibers,^[^ ^21^ ^]^ nanobowls,^[^ ^14^ ^]^ nanotoroids,^[^ ^22^ ^]^ and other ordered nanostructures.^[^ ^23^ ^]^ These characteristics render polypeptide‐based nanostructures highly suitable scaffolds for efficient drug delivery, offering new promise for the bottom‐up design of nanoscale delivery systems.^[^ ^24^, ^25^ ^]^

In addition, for developing next‐generation nanocarriers, multicompartmental nanocapsules are highly desired, featuring each compartment protected by a distinct and stable shell for versatile co‐encapsulation and sequential release.^[^ ^26^ ^]^ Unlike conventional spherical or cylindrical nanoparticles, their multi‐compartment structure enables the separate co‐encapsulation of multiple agents without cross‐contamination, which is essential for achieving enhanced performance in biomedical applications such as cancer therapy,^[^ ^27^ ^]^ confined enzymatic reactions,^[^ ^28^ ^]^ and tissue regeneration.^[^ ^29^ ^]^ For instance, the co‐delivery of two or more drugs that act synergistically can achieve greater therapeutic efficacy than the mere sum of each drug individually.^[^ ^30^ ^]^ Moreover, through co‐encapsulation, the sequential release of different chemotherapeutic agents can further enhance therapeutic synergy, thereby improving treatment outcomes while reducing toxicity.^[^ ^31^, ^32^ ^]^ Furthermore, in addition to co‐encapsulation and sequential release strategies, meticulous control over multicompartmental structure, capsule size, and the distribution uniformity is crucial for achieving the combined therapeutic effect. However, although many multicompartmental nanocapsules have been developed,^[^ ^33^, ^34^, ^35^ ^]^ most of these systems lack the ability to precisely control the morphology uniformity and co‐delivery of drugs with distinct solubilities and different release styles. Therefore, the development of uniform multicompartmental nanocapsules with separate hydrophobic and hydrophilic domains for co‐delivery of hydrophobic and hydrophilic agents, is still highly desired.

**Scheme 1:**
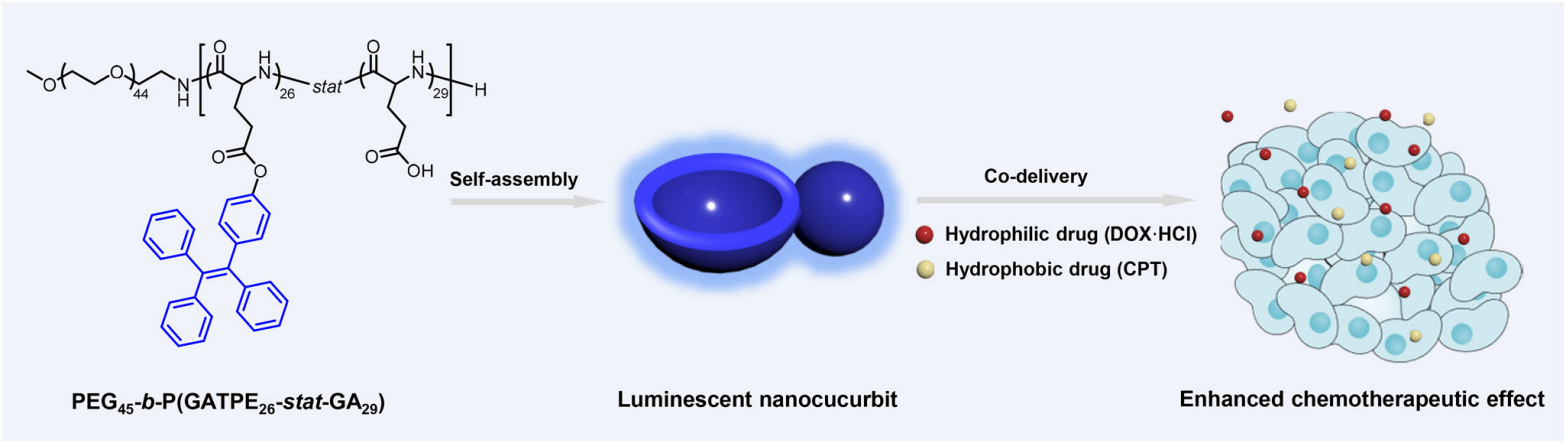
Schematic illustration of the luminescent nanocucurbits for spatiotemporal co‐delivery of hydrophilic and hydrophobic chemotherapeutic agents to tumor cells.

In this study, we propose a new self‐assembly methodology for creating luminescent nanoparticles featuring a unique cucurbit‐shaped topology. These nanocucurbits are self‐assembled from biodegradable polypeptide copolymer that are covalently modified with tetraphenylethylene (TPE) moieties, endowing the nanostructures with aggregation‐induced emission property. The luminescent nanocucurbits adopt an asymmetric nanostructure comprising two distinct compartments (a kippah vesicle and a multicompartment vesicle), making them ideal carriers for the simultaneous and efficient encapsulation of both hydrophilic and hydrophobic anticancer drugs (**Scheme** **1**). Herein, two widely used chemotherapeutic agents, doxorubicin hydrochloride (DOX) and camptothecin (CPT) were selected as model drugs due to their excellent anti‐tumor efficiency against various solid tumors. Those two drugs exhibited distinct solubility characteristics and different anticancer mechanisms.^[^ ^36^ ^]^ DOX is a hydrophilic compound and functions by inserting itself between DNA base pairs, triggering a cascade of biochemical reactions that ultimately lead to apoptosis in multiple tumor cells.^[^ ^37^ ^]^ In contrast, CPT, a pentacyclic quinoline alkaloid derived from plants, effectively eliminates cancer cells by interfering with the synthesis of DNA and RNA.^[^ ^38^ ^]^ Furthermore, it is well documented that the combination of DOX and CPT could improve the anticancer effect in various solid tumor models.^[^ ^39^, ^40^, ^41^ ^]^ The co‐delivery system shows differential drug release from independent compartment without mutual effect. In addition, the enhanced therapeutic effect of co‐loaded drug combination in nanocucurbits was demonstrated by the cytotoxicity promotion of the anticancer drug against human hepatocellular carcinoma (HepG2) cells.

## 2 Results and Discussion

### 2.1 Synthesis and Characterization of Copolymer

Well‐defined block copolymers were synthesized and characterized according to our previous work^[^ ^42^ ^]^ and the synthesis route is presented in Figure S1 (Supporting Information). Briefly, L‐glutamate‐*γ*‐benzyl *N*‐carboxyanhydride (Bz‐Glu NCA) monomer was prepared via the reaction between amino acids with triphosgen. Then, poly(ethylene glycol)_45_‐*b*‐poly(*γ*‐benzyl‐L‐glutamate)_55_ (PEG_45_‐*b*‐PBLG_55_) block copolymer was synthesized through the ring‐opening polymerization of Bz‐Glu NCA monomer with PEG_45_‐NH_2_ as a macroinitiator. The PEG_45_‐*b*‐PBLG_55_ copolymer was subsequently deprotected to obtain poly(ethylene glycol)_45_‐*b*‐poly(L‐glutamate)_55_ (PEG_45_‐*b*‐PGA_55_). Finally, the side chains of PEG_45_‐*b*‐PGA_55_ were partially modified with TPE to afford the grafted polypeptide poly(ethylene glycol)_45_‐*block*‐poly[(L‐glutamic acid‐TPE)_26_‐*stat*‐(L‐glutamic acid)_29_] [PEG_45_‐*b*‐P(GATPE_26_‐*stat*‐GA_29_)]. The synthetic details and the corresponding ^1^H NMR spectra of Bz‐Glu NCA, PEG_45_‐*b*‐PBLG_55_, PEG_45_‐*b*‐PGA_55_, and the final polymer PEG_45_‐*b*‐P(GATPE_26_‐*stat*‐GA_29_) are provided in Figures S2–S5 (Supporting Information). The degrees of polymerization (DPs) of PGA block and the grafting number of TPE are 55, and 26, as calculated from Figure S3–S5 (Supporting Information).

### 2.2 Self‐Assembling PEG_45_‐*b*‐P(GATPE_26_‐*stat*‐GA_29_) into Luminescent Nanocucurbits

**Figure 1:**
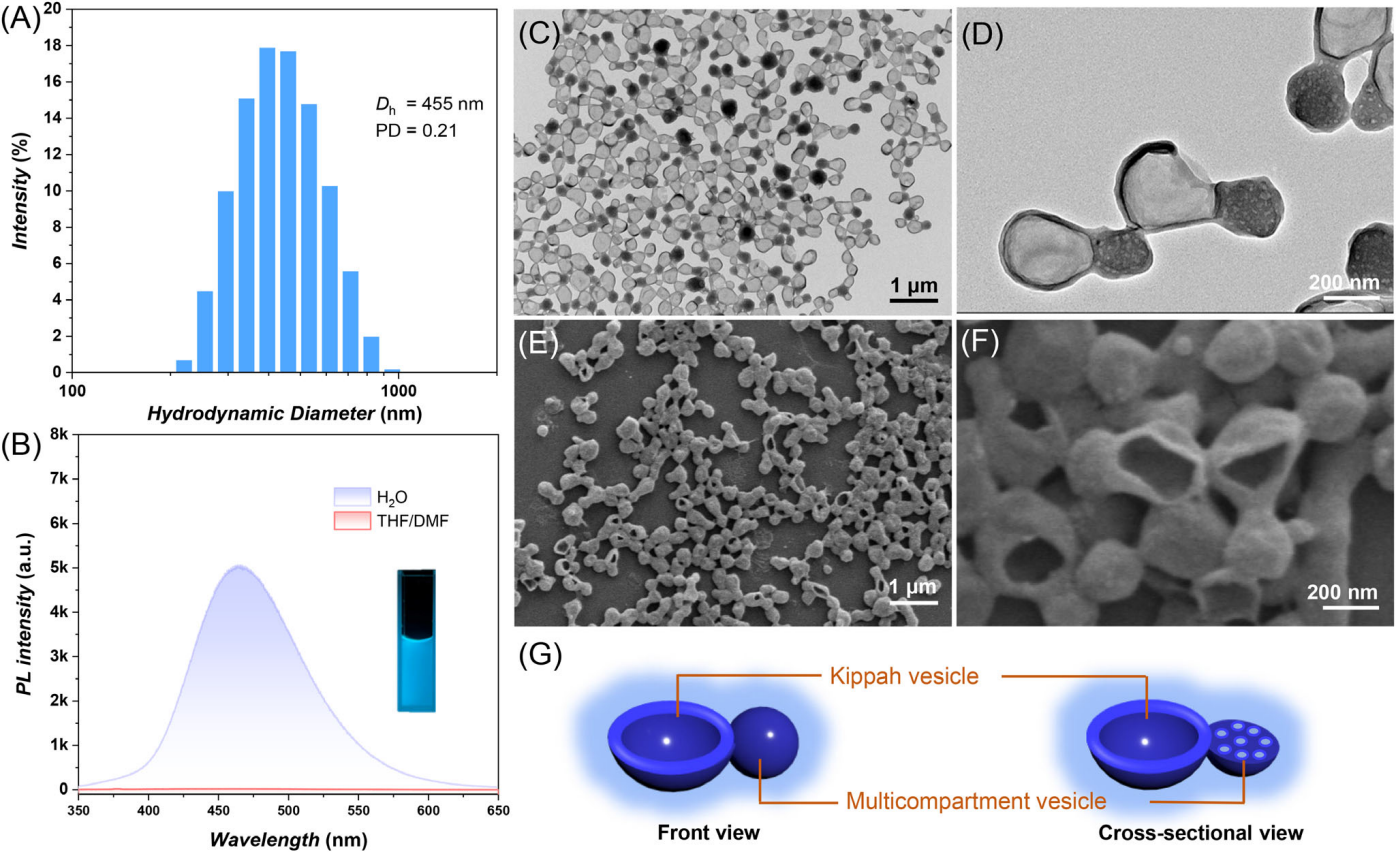
Characterization of the luminescent nanocucurbits self‐assembled from PEG_45_‐*b*‐P(GATPE_26_‐*stat*‐GA_29_) with THF/DMF as organic co‐solvents. A) Dynamic light scattering (DLS) analysis of the size variations of the nanocucurbits. B) Photoluminescence (PL) intensity of the nanocucurbits (*λ*_ex_ = 340 nm/*λ*_em_ = 462 nm). C,D) Transmission electron microscopy (TEM) images and E,F) scanning electron microscopy (SEM) images of nanocucurbits. G) Schematic diagram of a nanocucurbit consisting of a kippah vesicle and multicompartment vesicle (MCV) in front or cross‐sectional view.

The self‐assembly of PEG_45_‐*b*‐P(GATPE_26_‐*stat*‐GA_29_) was conducted via a typical solvent‐switch method. Briefly, the polypeptides were dissolved in a *N,N*‐dimethylformamide/tetrahydrofuran (DMF/THF) mixture (1.0 mg mL^−1^ polypeptides), followed by the gradual addition of three volume of deionized water using a peristaltic pump. Then the organic solvents were removed by dialyzing against deionized water for 48 h. The dynamic light scattering (DLS) study revealed that the self‐assemblies have a mean hydrodynamic diameter (*D*_h_) of 455 nm with a polydispersity (PD) of 0.21 (**Figure** **1**A). Unlike many other “hard” nanoparticles, such “soft” nanocucurbits are capable of turning themselves into smaller ones by deformation, thereby enabling passage through intercellular gaps along the interstitial flow and facilitating localized accumulation.^[^ ^43^, ^44^ ^]^ Instead of the extremely weak emission of PEG_45_‐*b*‐P(GATPE_26_‐*stat*‐GA_29_) in the discrete state, obvious fluorescence intensity can be detected by photoluminescence (PL) spectroscopy with an emission maximum of 462 nm and a bright blue fluorescence can be observed under UV irradiation with naked eyes (Figure 1B). This demonstrates that the TPE side groups in the restriction of intramolecular rotation mode, leading to the drastic aggregation after self‐assembly. Transmission electron microscopy (TEM) and scanning electron microscopy (SEM) were applied to reveal the morphology of the luminescent nanoparticles. Figure 1C,E confirmed that most of the nanoparticles with a relatively compact upper half and a collapsed hollow lower half asymmetric nanostructure. Figure 1D,F shows the magnified TEM and SEM images of the nanocucurbits, revealing combined assembly nanostructures  of kippah vesicles and multicompartment vesicles (MCVs).^[^ ^45^ ^]^ The statistical diameter of nanocucurbits is approximately 449 nm calculated from TEM images and approximately 431 nm calculated from SEM images (Figure S6, Supporting Information). Figure 1G shows the cucurbit‐shaped model of a nanocucurbit consisting of a kippah vesicle and MCV in front or cross‐sectional view.

The electrosteric stability of the luminescent nanocucurbits is a crucial criterion for further biomedical applications. These nanocucurbits exhibited excellent stability against extensive dilution with water. The *D*_h_ and PD showed no obvious change after diluting the nanocucurbits suspension to one‐tenth of the original concentration (*D*_h_ = 430 nm, PD = 0.213), indicating that these cucurbit‐shaped nanoparticles could maintain stability even at low concentration (Figure S7, Supporting Information). Additionally, these nanocucurbits exhibited excellent colloidal stability in aqueous solution for at least 7 days at room temperature (Figure S8, Supporting Information).

### 2.3 Revealing the Formation Mechanism of Luminescent Nanocucurbits

**Figure 2:**
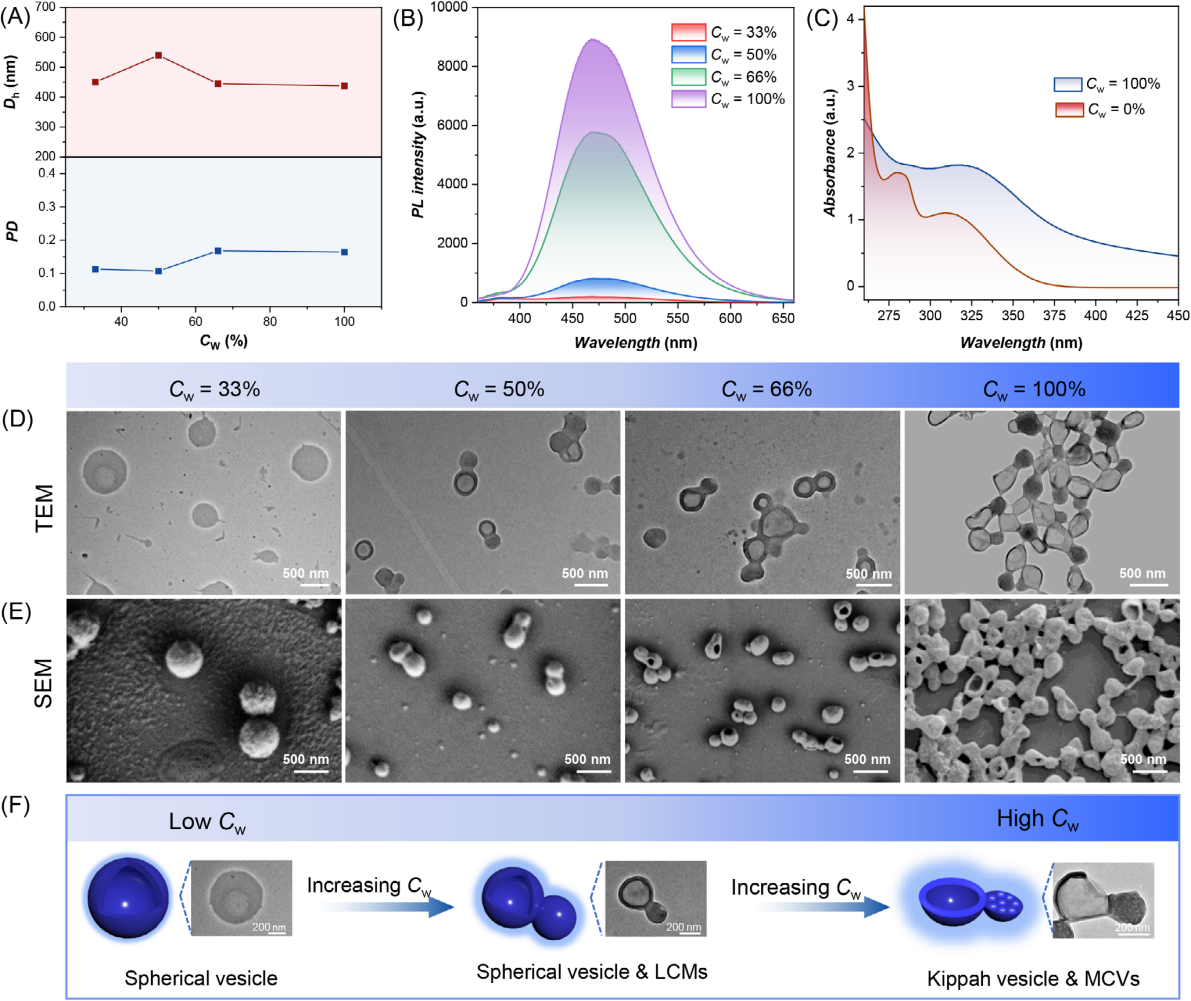
Formation process of luminescent nanocucurbits as monitored by A) DLS, B) PL (*λ*_ex_ = 340 nm/*λ*_em_ = 462 nm), C) Ultraviolet–visible (UV−vis) spectra, D) TEM, and E) SEM, On the top of electron microscopy images are different water content (*C*_w_). F) Illustration of the formation process.

Continuous addition of deionized water into the polypeptide solution decreased the miscibility of the polypeptides in the water/organic mixture, promoting hydrophobic self‐assembly. Therefore, we systematically investigated the effect of water content (*C*_w_) by tracing the formation process via TEM, SEM, DLS, PL and ultraviolet‐visible (UV−vis) spectroscopy. During gradually adding water to the THF/DMF solution of polypeptides, intermediate nanoparticle dispersions with different *C*_w_ were sampled before prior to analysis. When the *C*_w_ increased from 33% to 50%, DLS analysis showed that the *D*_h_ kept growing from 410 to 540 nm, indicating morphology transformation may occurr (**Figure** **2**A). Meanwhile, the PL intensity can be gradually detected by photoluminescence spectroscopy (Figure 2B). When the *C*_w_ continued to increase to 66%, the corresponding *D*_h_ decreased to 445 nm along with a rapidly increasing PL intensity. This significantly enhanced fluorescence signal can be attributed to the considerable aggregation of TPE groups in the self‐assembly of P(GATPE_26_‐*stat*‐GA_29_). Both *D*_h_ and PD became constant when the *C*_w_ increased to 100% but the PL intensity exhibited a continuous increase during the self‐assembly process. This trend suggests that the escape rate of THF, which swells the TPE groups on the polypeptide chains, accelerates significantly, thereby promoting the rapid aggregation of TPE groups. The inherent fluorescent features of cucurbit‐shaped nanoparticles were further characterized by UV–vis spectroscopy (Figure 2C). The absorption peak of nanocucurbits was located at 320 nm, which displays a slight red‐shift compared with that of PEG_45_‐*b*‐P(GATPE_26_‐*stat*‐GA_29_) dissolved in the DMF/THF co‐solvents.

Additionally, the intermediate nanoparticles at different *C*_w_ were analyzed by TEM and SEM studies (Figure 2D,E). When the *C*_w_ was 33%, large spherical vesicles were formed. In principle, an increase in *C*_w_ can lead to an increase in the corona repulsion within polymer vesicles, thereby enhancing the membrane tension by squeezing organic solvent “plasticizer” out of the vesicular membrane to overcome the energetic barriers.^[^ ^46^, ^47^ ^]^ In fact, as the *C*_w_ increases to 50%, large compound micelles (LCMs) were squeezed out from the large vesicles to form composite assemblies. With *C*_w_ continuing to increase and organic solvents kept flowing out, the membrane of large vesicle became relatively rigid and collapsed to form the kippah structure when a certain pressure was reached (*C*_w_ = 66%). Finally, when the residual organic solvents in the system were completely removed through dialysis for 48 h, relatively stable and evenly distributed nanocucurbits, that is, a combination of kippah vesicles and MCVs was formed when the *C*_w_ was 100%. It should be noted that the internal cavities of the MCVs in the composite assembly were evolved from the LCMs, since an increasing *C*_w_ led to the formation of inner hydrophobic membrane to ensure the nanostructures more stable.^[^ ^45^ ^]^ Compared to the LCMs, MCVs have a larger membrane surface that is exposed to water. Taken together, we proposed that the luminescent nanocucurbits originate from large spherical vesicles, undergoing outward extension of the vesicle membrane, giving spherical protrusions, eventually deforming into a combination of kippah vesicles and MCVs with a cucurbit‐like overall structures (Figure 2F).

### 2.4 Cytotoxicity and Hemolytic Assay

An ideal drug delivery carrier should possess optimal biocompatibility profiles at both cellular and hematological levels. We first evaluated the cytotoxicity of the nanocucurbits against HepG2 cells. Owing to their inherently biocompatible polypeptide composition, the relatively cell viability exceeded 90% in all groups incubated with nanocucurbits, demonstrating negligible cytotoxicity within the tested concentration range (Figure S9, Supporting Information). To assess hemocompatibility, a concentration‐gradient hemolysis assay was conducted. Notably, the hemolytic ratios remained below 2% across the tested concentration range (Figure S10, Supporting Information), aligning with the critical safety threshold of 5% hemolysis.^[^ ^48^, ^49^ ^]^ These collective findings substantiate the exceptional cytocompatibility and hemocompatibility of nanocucurbits, thereby validating their translational potential as next‐generation drug delivery vehicles.

### 2.5 Enhanced Cellular Uptake

Substantial evidence indicates that the interactions between nanomaterials and cells are critically governed by their physiochemical properties, such as size, shape, mechanical stiffness, surface charge, and chemical functionality.^[^ ^50^, ^51^ ^]^ To investigate the structure‐dependent internalization efficacy of this unique cucurbit‐shaped nanostructure, we engineered conventional spherical micelles (self‐assembled from identical polypeptide precursors) as comparative controls. Comprehensive characterization via DLS and TEM studies revealed monodisperse spherical micelles with a *D*_h_ of 231 nm (PD = 0.158) and typical solid core morphology (Figure S11, Supporting Information).

**Figure 3:**
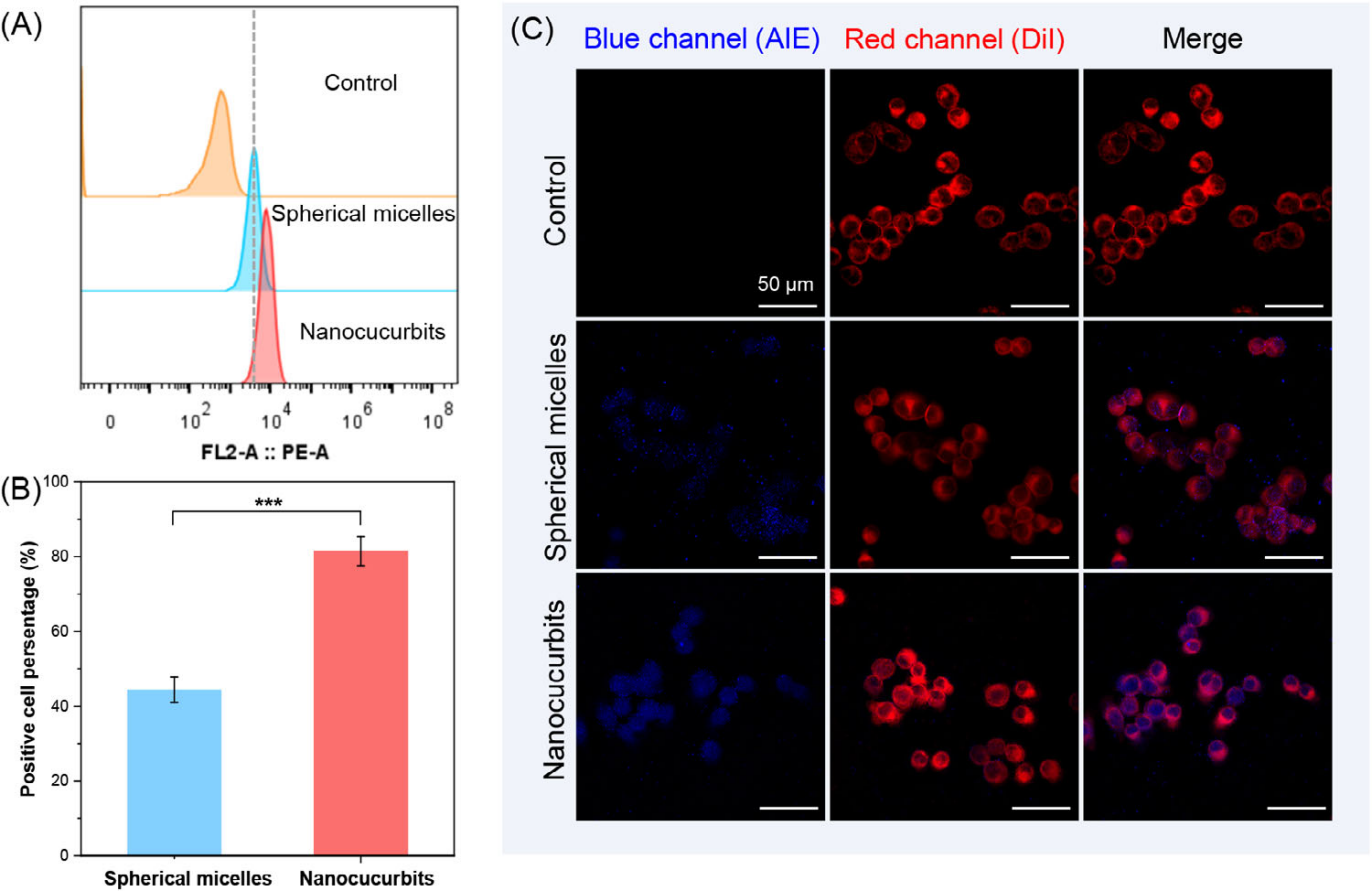
Cellular uptake and internalization of the luminescent nanocucurbits. A) Flow cytometry analysis of HepG2 cells treated by different formulations including PBS, spherical micelles, and nanocucurbits. (n = 3) B) Percentage of the Nile red (NR)‐positive cell population with reference to the negative control. Data are presented as mean ± s.d. (n = 3). The statistical analysis was performed with One‐way ANOVA analysis, *p < 0.05, **p < 0.01, ***p < 0.001. C) Confocal laser scanning microscopy (CLSM) images of HepG2 cells after incubation for 4 h with conventional spherical micelles and nanocucurbits. (n = 3; Scale bar = 100 µm).

Quantitative analysis of HepG2 cellular uptake was performed using Nile red (NR)‐labeled nanocucurbits with flow cytometric analysis. In comparison, conventional spherical micelles were also tested under the same condition. The percentage of red fluorescence‐positive cells after incubation was used to evaluate the cellular internalization efficiency. The results of flow cytometry (**Figure** **3A,B**; Figure S12, Supporting Information) showed that both the nanocucurbits and spherical micelles could enter the cells after 4 h of incubation, but the internalization efficiency was quite different. For the NR‐labelled nanocucurbits‐treated cells, ≈ 82% of HepG2 cells displayed red fluorescence, higher than that of spherical micelles (44%), thus unequivocally validating the geometric advantage of cucurbit‐shaped nanostructures in cellular internalization processes. Furthermore, the intracellular colocalization of nanocucurbits was further explored using confocal laser scanning microscopy (CLSM). Unlike the control group that only displayed red fluorescence derived from 1,1′‐dioctadecyl‐3,3,3′,3′‐tetramethylindocarbocyanine perchlorate (DiI)‐counterstained cell membranes, distinct blue fluorescence signals could be observed in HepG2 cells treated with nanocucurbits. Moreover, the intensity of blue fluorescence in HepG2 cells was much more concentrated and brighter than that of spherical micelles‐treated cells (Figure 3C). Statistical analysis of average blue fluorescence intensity was quantified and presented in Figure S13 (Supporting Information). The average fluorescence intensity of the nanocucurbits group was more than twice that of the spherical micelles group. It has been reported that nanoparticles with a size approximately ranging from 150 to 200 nm are primarily internalized via clathrin‐mediated and caveolin‐mediated endocytosis. Whereas for larger nanoparticles ranging from 250 nm to 3 µm, phagocytosis and micropinocytosis are considered the most efficient uptake pathways.^[^ ^52^, ^53^ ^]^ Therefore, the cellular uptake pathway for nanocucurbits (455 nm) may be phagocytosis and micropinocytosis. Moreover, the augmented endocytic uptake can be attributed to the structural anisotropy of the nanocucurbits, a geometric feature that confers enhanced surface‐to‐volume ratios mediating improved membrane interfacing.^[^ ^54^, ^55^ ^]^

### 2.6 Dual Drug Loading and Cumulative Release

Capitalizing on the excellent biocompatibility and enhanced cellular internalization of the luminescent nanocucurbits, we engineered a dual‐modal drug delivery platform capable of simultaneous hydrophobic/hydrophilic cargo encapsulation with spatiotemporally controlled release profiles. CPT and DOX were chosen as the hydrophobic and hydrophilic drugs to validate this structure‐function relationship. The co‐loading of CPT and DOX into nanocucurbits was implemented via a self‐assembled procedure, yielding compartment‐specific drug localization: CPT was localized within the hydrophobic dense membrane, while DOX occupied the hydrophilic lumen of the nanocucurbit.

**Figure 4:**
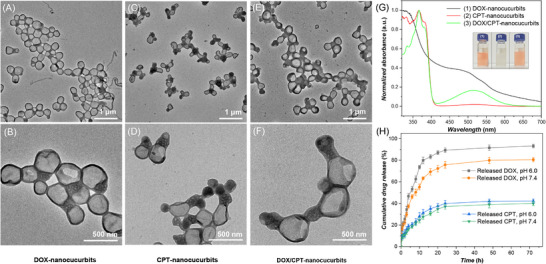
Drug loading and cumulative release profiles of DOX‐nanocucurbits, CPT‐nanocucurbits, and DOX/CPT‐nanocucurbits. A,B) TEM images with different magnification of DOX‐nanocucurbits. C,D) TEM images with different magnification of CPT‐nanocucurbits. E,F) TEM images with different magnification of DOX/CPT‐nanocucurbits. G) UV–vis spectra and the corresponding photographs of nanocucurbits suspensions loaded with different drugs. H) Cumulative drug release of CPT and DOX from DOX/CPT‐nanocucurbits in different pH values (n = 3).

The resulting drug‐loaded nanocucurbits were first characterized by DLS and Figure S14 (Supporting Information) summarized the mean hydrodynamic diameters. The size of DOX loaded nanocucurbits (DOX‐nanocucurbits) and DOX and CPT co‐loaded nanocucurbits (DOX/CPT‐nanocucurbits) were slightly larger than those of the blank nanocucurbits (455 nm), being 474 and 512 nm respectively. This indicated that the presence of DOX in the hydrophilic lumen increased the volume. Conversely, CPT loaded nanocucurbits (CPT‐nanocucurbits) showed the smallest diameter, which might be attributed to the incorporation of hydrophobic drugs making the amphiphilic polypeptides form a more compact structure.^[^ ^36^ ^]^ Besides, the corresponding TEM images of nanocucurbits loaded with different drugs were shown in **Figure** **4**A–F. It can be seen that different drug‐loaded assemblies retain a cucurbit‐like morphology. Specifically, DOX‐nanocucurbits display an expanded structure within the hydrophilic lumens of kippah vesicles, whereas CPT‐nanocucurbits adopt a more compact and hydrophobic membrane structure within multicompartment vesicles. Moreover, assemblies co‐encapsulating both DOX and CPT exhibit a slightly increased overall size. Furthermore, UV–vis spectra and the embedded photos of the suspensions of DOX‐nanocucurbits, CPT‐nanocucurbits, and DOX/CPT‐nanocucurbits, are presented in Figure 4G. The characteristic peak observed at either 485 nm (aqueous solution) or 515 nm (after lysing in THF/DMF mixture) is attributed to DOX, while the peak at 367 nm corresponds to CPT, confirming successful co‐encapsulation. Calibration curves established drug loading efficiencies of 26 wt% for CPT and 15 wt% for DOX (Figures S15 and S16, Supporting Information),^[^ ^56^, ^57^ ^]^ highlighting the system's enhanced affinity for hydrophobic drug payloads over hydrophilic counterparts.

A hallmark of engineered drug delivery systems lies in their capacity to orchestrate controlled release kinetics, thereby prolonging therapeutic exposure windows while mitigating off‐target toxicity.^[^ ^58^, ^59^ ^]^ To elucidate the compartment‐specific release mechanisms of this dual‐drug platform, two different pH values (7.4 and 6.0) were selected to mimic normal physiological environment and the slightly acidic tumor microenvironment. As shown in Figure 4H, DOX exhibits a higher release rate under acidic conditions than under neutral conditions, achieving cumulative releases of 93% and 81% at 72 h, respectively. Whereas CPT release exhibits minimal pH dependence, showing comparable cumulative release of 42% (pH 6.0) and 40% (pH 7.4) after 72 h. The accelerated drug‐release rate at pH 6.0 was mainly due to the presence of ionizable primary amine group of DOX (Figure S17, Supporting Information). This differential release kinetics primarily stems from the distinct physicochemical properties of the two drugs and their specific interactions with the components of our delivery system. For CPT, as a highly hydrophobic molecule, it is predominantly encapsulated within the hydrophobic bilayer membrane of the nanocucurbits via strong hydrophobic interactions. Release requires the gradual degradation/diffusion of the carrier material or significant partitioning into the aqueous environment, which is a relatively slow process.^[^ ^60^ ^]^ In contrast, DOX exhibits amphiphilic properties due to its ionizable amine group and is preferentially localized in the hydrophilic lumen of the nanocucurbits. In an aqueous environment, DOX·HCl undergoes rapid deprotonation, facilitating faster diffusion‐driven release.^[^ ^61^ ^]^

Moreover, the intentionally slower release of CPT compared to the faster release of DOX confers several crucial advantages for effective combination therapy, particularly considering their distinct mechanisms of action and optimal timing. Studies have demonstrated that the sequence of release (e.g., a DNA‐damaging agent like DOX followed by a topoisomerase I inhibitor like CPT) can significantly impact synergy.^[^ ^62^, ^63^ ^]^ In this co‐delivery system, DOX is released relatively quickly, acting as the “priming” agent. It intercalates into DNA, causes DNA damage, and sensitizes the HepG2 cells. Whereas CPT is released more gradually and persistently. As HepG2 cells are arrested by the inhibition of DOX, they become more vulnerable to CPT. Besides, the sustained release ensures prolonged exposure to CPT, effectively targeting cells as they progress through or attempt to repair after DOX‐induced damage. The simultaneous high peak concentrations of both drugs might sometimes lead to antagonistic effects or overwhelming toxicity. The staggered release profile helps mitigate this risk by preventing unnecessarily high concurrent local concentrations immediately upon arrival at the tumor cells. Collectively, this different release behavior of DOX and CPT indicates that the co‐delivery system provides a possibility of their synergistic effect.

### 2.7 Synergistic Chemotherapeutic Effects

**Figure 5:**
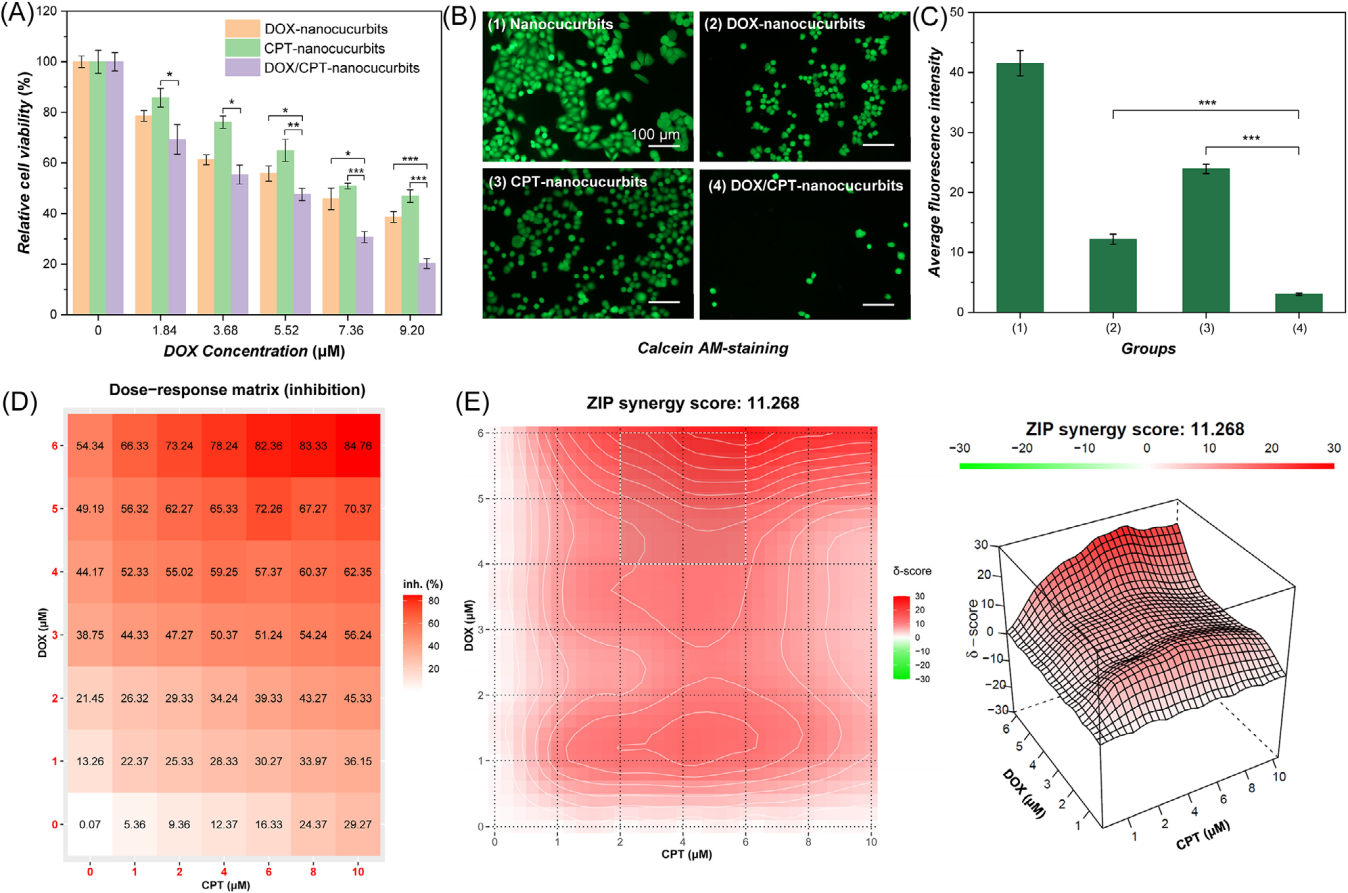
Evaluation of the synergistic effect. A) Cytotoxicity of nanocucurbits loaded with different drugs against HepG2 cells at various DOX concentrations. The values are expressed as mean ± s.d. (n = 3). Statistical significance was determined by one‐way ANOVA: (*) for *p* < 0.05, (**) for *p* < 0.01, and (***) for *p* < 0.001. B) Representative fluorescence images of HepG2 cells co‐stained with calcein acetoxymethy ester (Calcein‐AM, live cells, green) after different treatments. Scale bar: 100 µm. C) Statistical analysis of average green fluorescence intensity in each group (n = 3). Data in C are presented as means ± SD (n = 3). P values were calculated by one‐way ANOVA with a Tukey's test. *p < 0.05, **p < 0.01, ***p < 0.001. D) Dose‐response matrix. The concentration of DOX or CPT refers to the concentration of DOX within the DOX‐nanocucurbits or CPT‐nanocucurbits. E) Synergy distribution and the Zero interaction potency (ZIP) scoring to eliminate the synergy results.

To systematically assess the therapeutic advantage of combination therapy, a CCK‐8 assay was first carried out to evaluate the cytotoxicity of CPT‐nanocucurbits, DOX‐nanocucurbits, and DOX/CPT‐nanocucurbits. As shown in **Figure** **5**A, all three groups showed concentration‐dependent cytotoxicity. There is a significant difference between CPT‐nanocucurbits or DOX‐nanocucurbit and DOX/CPT‐nanocucurbits (DOX] = 9.20 µM), and the inhibition rates were 53%, 61%, and 80%, respectively. This result demonstrates that the combination of drug treatments demonstrated a significantly enhanced inhibition when compared to a single drug.

Additionally, the calcein acetoxymethy ester (Calcein‐AM), and Hoechst 33342 staining assays were investigated to further confirm the anticancer efficiency of the drug‐loaded nanocucurbits. As shown in Figure 5B,C, HepG2 cells incubated solely with nanocucurbits exhibited minimal cell death, as evidenced by the presence of green fluorescence. This finding indicated that the nanocucurbits demonstrate excellent good cytocompatibility at this concentration. Notably, treatment with single drug‐loaded nanocucurbits (DOX or CPT alone) induced moderate cytotoxicity, with partial dead cells be observed within the field of view. In stark contrast, co‐delivery of DOX/CPT via nanocucurbits exhibited near‐complete cancer cell eradication, as revealed by the red fluorescence, which conforms to the results of the CCK‐8 assay. Besides, we also repeated the live cell staining assay using Hoechst 33342 to verify this result (Figure S18, Supporting Information). In principle, Hoechst 33342 can penetrate the cell membrane and bind to the minor groove of dsDNA sequences rich in A/T, thereby achieving staining of the cell nucleus in living cells. Normal living cells exhibit uniform blue fluorescence. Early apoptotic cells, owing to increased permeability of the cell membrane, display dense staining. Late apoptotic or necrotic cells, as dsDNA undergoes degradation, show diminished blue fluorescence. Accordingly, the inhibition rate of HepG2 cells follows the following pattern: DOX/CPT‐nanocucurbits > DOX‐nanocucurbits > CPT‐nanocucurbits. Taken together, these findings unequivocally establish DOX/CPT‐nanocucurbits as a potent combination chemotherapeutic platform, capable of achieving synergistic tumor cell elimination through mechanistically complementary drug actions.

Moreover, to maximize the synergistic effects of the two drugs, a more comprehensive cytotoxicity study using various concentrations and ratios of DOX and CPT is performed. The expected drug combination responses were calculated based on Zero interaction potency (ZIP) reference model using SynergyFinder.^[^ ^64^ ^]^ Deviations between observed and expected responses with positive and negative values denote synergy and antagonism respectively. The analysis revealed that the two drugs exhibited synergistic effects over most of the dose range (synergy score > 0). Notably, the highest synergy score (11.268) was observed when DOX and CPT were administered at a dose ratio of 1.25:1(Figure 5D,E).

### 2.8 In Vivo Biodistribution

To evaluate the biodistribution of the nanocucurbits in vivo, a near‐infrared fluorescent dye, indocyanine green (ICG), was employed to label the nanocucurbits, thereby enhancing tissue penetration and reducing background fluorescence noise in the imaging study. For assessing the biodistribution and metabolism of the nanocucurbits, free ICG and ICG labelled nanocucurbits (ICG‐nanocucurbits) were administered via tail vein injection. Continuous monitoring of ICG fluorescence at various time points showed that free ICG had a short circulation time in the bloodstream, with weak fluorescence observed beyond 8 h (Figure S19A, Supporting Information). In contrast, ICG‐nanocucurbits with a zeta potential of −23.5 mV, effectively avoided macrophage phagocytosis, thereby extending their circulation time in the bloodstream.^[^ ^16^ ^]^ In a separate experiment, mice were subsequently sacrificed at 8 h post‐injection, and the major organs were harvested for ex vivo fluorescence evaluation (Figure S19B,C, Supporting Information). Consistent with the in vivo imaging results, ICG‐nanocucurbits exhibited a high degree of accumulation in the liver and kidney compared to free ICG via the enhanced permeability and retention (EPR) effect. This prolonged circulation time and enhanced tissue enrichment effect offer promising potential for the subsequent treatment of in situ liver cancer or subcutaneous tumors. Building upon the promising results presented here, future studies will involve the functionalization of these luminescent nanocucurbits with targeting agents to enhance their tumor‐homing capability, thereby paving the way for synergistic tumor therapy.

## 3 Conclusion

In summary, geometry‐engineered luminescent nanocucurbits were constructed through the precise self‐assembly of AIE fluorophore‐conjugated polypeptides. These nanocucurbits exhibit anisotropic nanostructures and possess intrinsic fluorescence, enabling in situ cell imaging capabilities. Flow cytometric analysis and CLSM observations systematically confirmed that these asymmetric nanocucurbits achieved approximately two‐fold higher cellular internalization compared to their spherical counterparts, which can be attributed to their increased surface‐to‐volume ratios mediating membrane interaction. Furthermore, capitalizing on their compartmentalized nanostructure, the nanocucurbits can serve as a co‐delivery platform enabling spatially resolved encapsulation with hydrophilic DOX within the aqueous lumen and hydrophobic CPT in the dense regions. This structural feature allows for differential release of these two types of anticancer drugs with distinct physicochemical properties, thereby minimizing toxicity and adverse effects while supporting the potential for synergistic therapy. Dual drug‐loading in separate modules exhibits a higher anticancer cell effect than the single drug formulations. DOX/CPT‐nanocucurbits brought forth the enhanced chemotherapeutic effect against HepG2 cells. The concept of spatiotemporal co‐delivery strategy offers transformative insights into the development of next‐generation therapeutic platforms, providing critical insight into the design of versatile nanocarriers for tumor therapy.

## 4 Experimental Section

### Synthesis of PEG_45_‐b‐P(GATPE_26_‐stat‐GA_29_) Copolymer

Bz‐Glu NCA, PEG_45_‐*b*‐PBLG_55_, and PEG_45_‐*b*‐PGA_55_ were synthesized according to the previously reported methods.^[^ ^42^ ^]^ PEG_45_‐*b*‐PGA_55_ (0.500 g, 0.058 mmol) was dissolved in 2.0 mL of anhydrous DMF in a round‐bottomed flask. Then DMAP (0.021 g, 0.175 mmol) and TPE‐OH (0.305 g, 0.876 mmol) were added together with another 3.0 mL of anhydrous DMF. The mixture was stirred at 50 °C for 96 h. The solution was precipitated in diethyl ether for three times and the centrifuged residue was dried under vacuum for 24 h. The obtained solid was further purified by redissolving in DMF and dialyzing against deionized water, yielding an off‐white powder after lyophilization.

### Preparation of Luminescent Nanocucurbits

PEG_45_‐*b*‐P(GATPE_26_‐*stat*‐GA_29_) was dissolved in the DMF/THF mixture with an initial concentration of 1.0 mg mL^−1^, where the volume fraction of DMF in the DMF/THF mixture (*f*_DMF_) was 20%. Then deionized water (*V*_water_/*V*_mixture solvent_ = 3/1) was added into the solution dropwise under stirring at room temperature. The mixture solvent was removed by dialyzing against deionized water for 2 days. Under this condition, assemblies with cucurbit‐shaped topology can be formed based on the previous work.^[^ ^42^ ^]^

### Tracing the Formation Process of the Nanocucurbits

PEG_45_‐*b*‐P(GATPE_26_‐*stat*‐GA_29_) copolymer was dissolved in DMF/THF mixture and self‐assembled with the same solvent‐switching method described above. The samples with different water contents (33, 50, 66 and 100%) used for DLS, photoluminescence and TEM analysis.

### Preparation of Spherical Micelles

PEG_45_‐*b*‐P(GATPE_26_‐*stat*‐GA_29_) was dissolved in the DMF solution with an initial concentration of 1.0 mg mL^−1^. Then deionized water (*V*_water_/*V*_mixture solvent_ = 3/1) was added into the solution dropwise under stirring at room temperature. The mixture solvent was removed by dialyzing against deionized water for 2 days.

### Encapsulation of DOX in Nanocucurbits

PEG_45_‐*b*‐P(GATPE_26_‐*stat*‐GA_29_) copolymer (5.0 mg) was dissolved in the DMF/THF mixture with an initial concentration of 1.0 mg mL^−1^, where the volume fraction of DMF in the DMF/THF mixture was 20%. Then 15 mL deionized water containing DOX (3.75 mg) was added into the solution dropwise under stirring at room temperature in the dark. The unloaded DOX was removed by dialysis in tris buffer (0.01 M; pH 7.4) at room temperature with 300 rpm stirring, still under dark conditions.

### Encapsulation of CPT in Nanocucurbits

PEG_45_‐*b*‐P(GATPE_26_‐*stat*‐GA_29_) copolymer (5.0 mg) and CPT were dissolved in the DMF/THF mixture with an initial concentration of 1.0 mg mL^−1^, where the volume fraction of DMF in the DMF/THF mixture was 20%. Then 15 mL deionized water was added into the solution dropwise under stirring at room temperature in the dark. The CPT‐loaded nanocucurbits was obtained after purification via ultrafiltration.

### Co‐encapsulation of DOX and CPT in Nanocucurbits

PEG_45_‐*b*‐P(GATPE_26_‐*stat*‐GA_29_) copolymer (5.0 mg) and CPT were dissolved in the DMF/THF mixture with an initial concentration of 1.0 mg mL^−1^, where the volume fraction of DMF in the DMF/THF mixture was 20%. Then 15 mL deionized water containing DOX (3.75 mg) was added into the solution dropwise under stirring at room temperature in the dark. The DOX and CPT co‐loaded nanocucurbits were centrifuged and washed three times to remove the CPT and DOX that had adsorbed on the surface. The CPT content was quantified by lysing a certain amount of nanoparticles in 1 mL of THF/DMF mixture (4:1 v/v). The loading capacities of DOX and CPT were calculated using UV‐vis spectroscopy at 485 and 367 nm, respectively.

### Cumulative Drug Release

The cumulative drug release of DOX and CPT was achieved by the following protocol. Namely, DOX and CPT co‐loaded nanocucurbits were transferred into a dialysis bag (cutoff molecular weight: 3500), which was then put into the release medium (PBS buffer with tween 80, pH 7.4 or 6.0) and the release process in a constant temperature shaking table (100 rpm, 37 °C). At different time intervals, the environmental buffer solution was replaced with fresh PBS containing tween 80, and the absorbances of the released DOX and CPT in the removed PBS solution was quantitatively monitored by UV‐vis spectroscopy. The corresponding concentrations of DOX and CPT were determined using a calibration curve where DOX showed its maximum absorbance (483 nm) and CPT showed its maximum absorbance (367 nm), respectively. Finally, the accumulative ratios of the released DOX were calculated as a function of time. Three parallel experiments were set up to take the average value.

### Cell lines and Culture

HepG2 cells were acquired from the cell bank of the Committee on Type Culture Collection of the Chinese Academy of Sciences (CTCC, Shanghai, China, CSTR:19375.09.3101HUMSCSP510). HepG2 cells were cultured with high glucose DMEM supplemented with 10% fetal bovine serum (FBS) and 1% penicillin/ streptomycin and cultured at 37 °C in a 5% CO_2_ humidified environment. The media used to culture HepG2 cells was changed every two days and the cells were digested using trypsin‐EDTA, which was then re‐suspended in a fresh complete medium before plating.

### Cellular Uptake and Colocalization

Initially, HepG2 cells were seeded in confocal dishes at a density of 4 × 10^5^ and cultured in a 5% CO_2_ incubator overnight at 37 °C. Subsequently, the HepG2 cells were incubated with nanocucurbits or spherical micelles at the final concentration of 125 µg mL^−1^ for 2 h, followed by washing with PBS for 3 times. Next, the cells were fixed at 4 °C with 4% paraformaldehyde for 30 min followed by washing with PBS for 3 times. Then, HepG2 cells were labeled with 10 µM DiI, which is a red fluorescent probe for cell membrane. Finally, the fluorescence was observed with CLSM under a 40 × objective.

### Evaluation of the Synergistic Effect of DOX/CPT‐nanocucurbits toward Tumor Cells

The synergistic effect of DOX and CPT co‐loaded nanocucurbits was further evaluated by CCK‐8 assay, Calcein‐AM or Hoechst 33342 staining. In brief, HepG2 cells were seeded into 96‐well plates at a density of 8 × 10^3^ per well and cultured in a 37 °C, 5% CO_2_ incubator overnight. Then, the culture media were replaced with 100 µL fresh media containing sterile PBS, DOX‐nanocucurbits, CPT‐nanocucurbits, and DOX/CPT nanocucurbits, respectively. After 24 h of incubation, the amounts of viable cells were measured by CCK‐8 assay according to the manufacturer's instructions.

Additionally, live cell staining assays were also carried out to visualize the synergistic therapeutic effect on tumor cells. In brief, HepG2 cells were seeded in 24‐well plates at a density of 1 × 10^5^ per well and cultured in a 37 °C, 5% CO_2_ incubator overnight and treated with the same way as described above. After 24 h of incubation, HepG2 cells were washed with PBS for 3 times and stained with Calcein AM for 20 min or Hoechst 33342 for 5 min. Finally, the cells were observed using a Nikon inverted microscope ECLIPSE Ts2 under an inverted fluorescence microscope (Nikon eclipse Ts2).

### Statistical Analysis

Data in bar graphs were presented as mean ± standard deviation (SD). Statistical analyses were conducted using OriginPro software (version 2021). Comparisons among multiple groups were performed using One‐way analysis of variance (ANOVA), followed by Tukey's test, and a *p*‐value less than 0.05 was considered to be statistically significant.

### In Vivo Biodistribution

Female NOG mice (5‐6 weeks old, weighing 18–20 g) were procured from GemPharmatech Co., Ltd. (Guangdong, China). Approval for all animal experiments was granted by the Institutional Animal Care and Use Committee of Servicebio (Approval No. 2025200). The NOG mice received intravenous injections via the tail with free indocyanine green (ICG) and ICG‐labelled nanocucurbits (ICG: 2 mg kg^−1^) in order to assess their in vivo biodistribution. At different time points following the injection (0 h, 0.5, 1, 2, 4, and 8 h), the mice were observed and imaged using an IVIS Spectrum (IVIS Lumina LT Series; PerkinElmer) with excitation at 780 and an 831 nm filter to collect the fluorescence emission of ICG. Following the completion of the imaging experiment, the mice were humanely euthanized, and the major organs were carefully excised for ex vivo fluorescence imaging.

## Conflict of Interest

The authors declare no conflict of interest.

## Supporting information



Supporting Information

## Data Availability

The data that support the findings of this study are available from the corresponding author upon reasonable request.
