# Supplementary material for: Luminescent Nanocucurbits Enable Spatiotemporal Co‐Delivery of Hydrophilic and Hydrophobic Chemotherapeutic Agents
Source: Adv Sci (Weinh). 2025 Sep 23;12(46):e09782. doi: 10.1002/advs.202509782 (PMC12697897; doi:10.1002/advs.202509782)
Supplement: Supplementary file 1 — Supporting Information [file ADVS-12-e09782-s001.pdf]

Supporting Information

Luminescent Nanocucurbits Enable Spatiotemporal Co-Delivery of Hydrophilic and Hydrophobic Chemotherapeutic Agents

Ping Wei, Yunshan Ding, Shangning Liu, Jinhui Jiang,* and Jinghua Chen**

Table of Contents

Experimental Section 2
  1. Materials 2
  2. Characterization 3
  3. Methods 4
Scheme and Figures 5
Reference 14

Experimental Section

1. Materials

Triphosgene (99%), (−)-*α*-pinene (98%), L-glutamic acid *γ*-benzyl ester (98%), 4-(1,2,2-triphenylvinyl)phenol (TPE-OH, 97%), trifluoroacetic acid (TFA, 98%), *N,N'*-diisopropylcarbodiimide (DIC, 98%), 4-dimethylaminopyridine (DMAP, 99%), poly(ethylene glycol) amine (PEG_{45}-NH_2, M_w = 2000 g/mol), and indocyanine green (ICG) were purchased from Aladdin Bio-Chem Technology Co., Ltd. (Shanghai, China). Hydrogen bromide (HBr/CH_3COOH, 33 wt% in acetic acid) was purchased from Beijing Innochem Science & Technology Co., Ltd. $CDCl_3$ and DMSO-d_6 was purchased from J&K Scientific Ltd. Tetrahydrofuran (THF), *n*-hexane, anhydrous *N, N*-dimethylformamide (DMF), methanol, acetone and diethyl ether were purchased from Sinopharm Chemical Reagent Co., Ltd. (SCRC, Shanghai, China) and used without further purification. THF was dried with sodium to remove traces of water before use. Doxorubicin hydrochloride (DOX·HCl, 98%) and camptothecin (CPT, 97%) were purchased from Macklin Co., Ltd. (Shanghai, China). Cell Counting Kit-8, Calcein/PI viability assay Kit, Hoechst 33342 staining solution for live cells, 4% paraformaldehyde, and 1,1-dioctadecyl-3,3,3,3-tetramethylindocarbocyanine perchlorate (DiI) were purchased from Beyotime Biotechnology Co., Ltd. Penicillin and streptomycin, trypsin, and Dulbecco's modified eagle medium (DMEM) were purchased from Thermo Fisher Scientific Co., Ltd. (Shanghai, China). Fetal bovine serum (FBS) and phosphate-buffered saline (pH 7.4, sterile) were purchased from Titan Scientific Co., Ltd (Shanghai, China).

2. Characterization

2.1 Dynamic Light Scattering (DLS)

The apparent hydrodynamic diameters (D_h) and polydispersity (PD) of the self-assemblies were determined by DLS using a ZETASIZER Nano series instrument (Malvern Instruments ZS 90) at a fixed scattering angle of 90º. Data processing was carried out using cumulant analysis of the experimental correlation function and calculated from the computed diffusion coefficients using the Stokes-Einstein equation. Each reported measurement was conducted for three runs.

2.2 Transmission Electron Microscopy (TEM)

The aqueous solution of the nano-objects (8.0 μL, 0.1 mg/mL) was dropped onto a copper grid covered by carbon layer and dried at 25 ºC. Images were recorded on a JEOL JEM-2100F instrument at 200 kV equipped with a Gatan 894 Ultrascan 1k CCD camera.

2.3 Scanning Electron Microscopy (SEM)

SEM was utilized to observe the surface morphologies of the self-assemblies. To prepare SEM samples, a drop of solution was spread on a silicon wafer at 25 ºC and left until dryness. The samples were coated with gold and viewed by a Hitachi Regulus 8100 electron microscopy operated at 3 kV. The images were recorded by a digital camera.

2.4 Fluorescence Spectroscopy

Fluorescence experiments were carried out *via* a Lumina fluorescence spectrometer (ThermoFisher).

2.5 UV-vis Spectroscopy

The UV-vis spectra were acquired using a UV759S UV-vis spectrophotometer (Shanghai Precision & Scientific Instrument Co., Ltd.). All the samples were analyzed using quartz cuvettes.

2.6 Inverted Fluorescence Microscopy

The fluorescent-based Live/Dead assays were performed on a Nikon inverted microscope ECLIPSE Ts2.

2.7 Confocal Laser Scanning Microscopy (CLSM)

Cell imaging experiments were carried out on a Nikon Ti2-E A1 Microscope. The red fluorescence signal of DiI was excited by an argon ion laser at 561 nm, and the blue fluorescence of TPE was excited at 405 nm, respectively. Then all images acquired were exported by using the NIS-Elements AR software.

3. Methods

3.1 Synthesis of L-glutamate *γ*-benzyl *N*-carboxyanhydride (Bz-Glu NCA) Monomer

Bz-Glu NCA monomer was synthesized according to our previously reported method.[1] The resulting white powder was further dried in a vacuum oven at 25 °C. Yield: ~78%.

3.2 Synthesis of PEG_{45}-*b*-$PBLG_{55}$

PEG_{45}-NH_2 (0.400 g, 0.200 mmol) and Bz-Glu NCA (3.159 g, 12.00 mmol) were dissolved in 10 mL of anhydrous DMF in a round-bottomed flask and stirred under high vacuum at 35 °C for 48 h. The solution was precipitated three times in methanol and the centrifuged residue was further purified by Soxhlet extraction with methanol as the solvent, yielding an off-white solid.

3.3 Synthesis of PEG_{45}-*b*-PGA_{55}

PEG_{45}-*b*-$PBLG_{55}$ (2.000 g) was dissolved in TFA (15 mL) in a 50 mL round-bottomed flask. An excess of HBr solution (5 mL, 33% in acetic acid) was added into the reaction flask. The reaction solution was stirred at room temperature for 5 h. Then the solution was precipitated in acetone for three times and the centrifuged residue was further purified by Soxhlet extraction with acetone as the solvent, yielding a beige solid.

Scheme and Figures

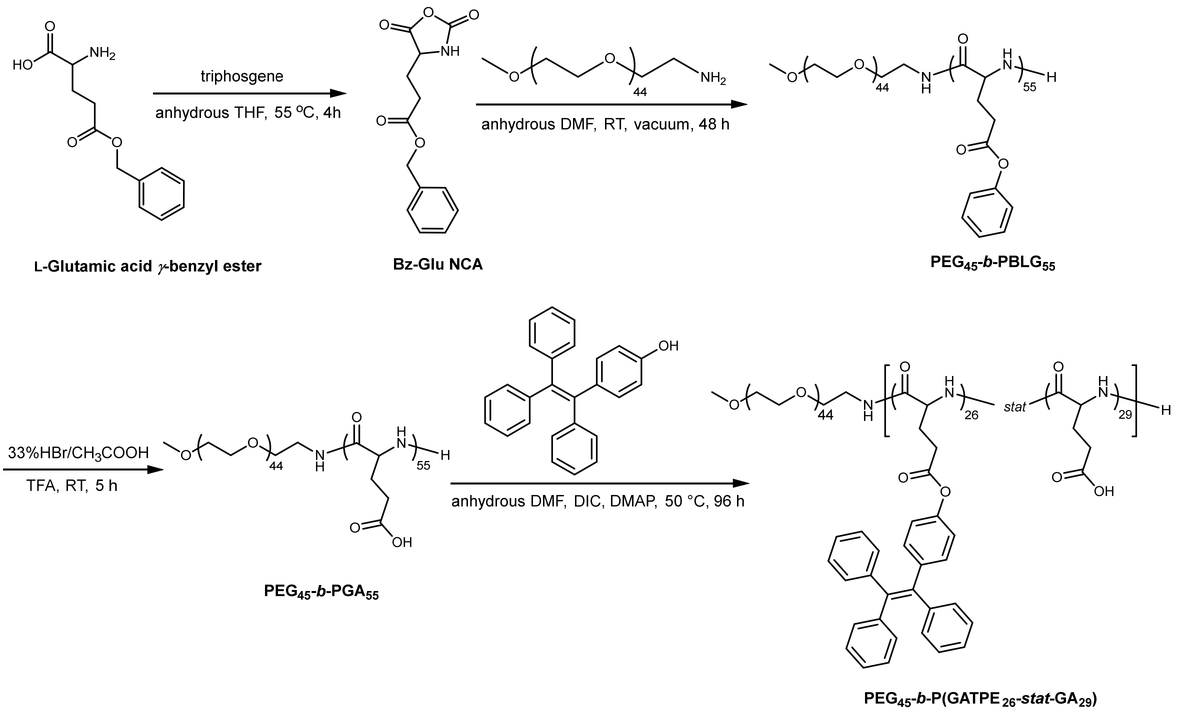

Figure S1. Synthesis route of monomers and PEG45-*b*-P(GATPE26-*stat*-GA29) copolymer.

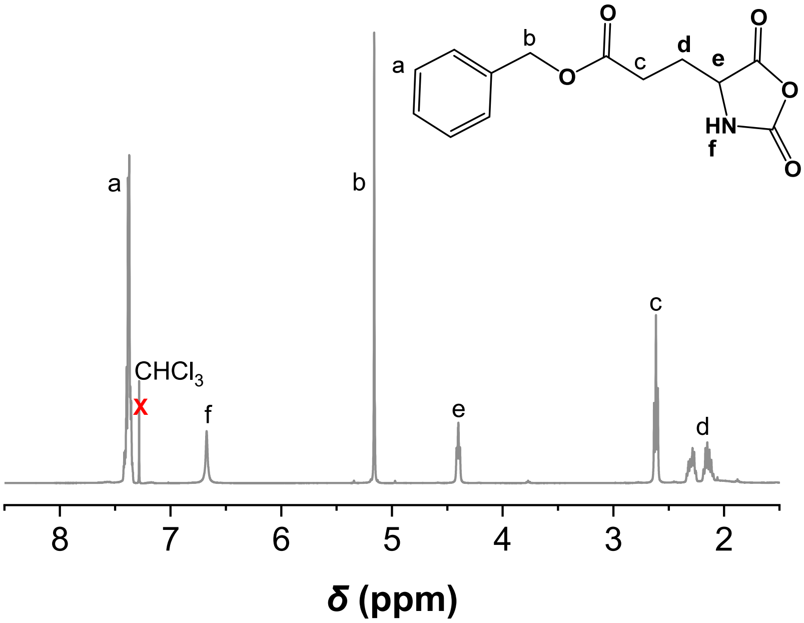

Figure S2. ^{1}H NMR spectrum of Bz-Glu NCA in $CDCl_3$.

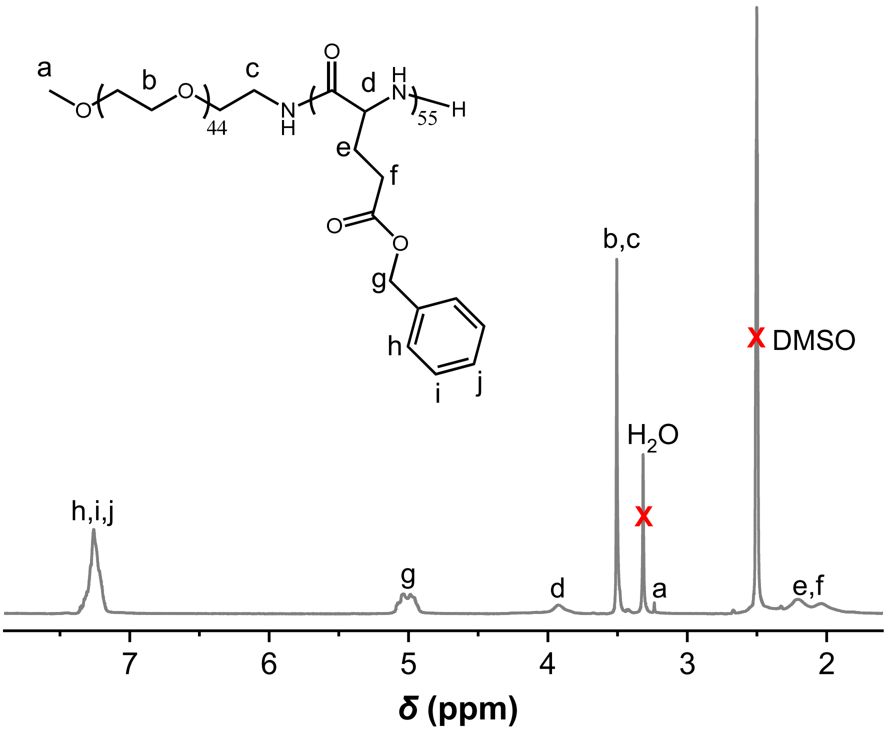

Figure S3. ^{1}H NMR spectrum of PEG$_{45}$-*b*-PBLG$_{55}$ in DMSO-*d*$_6$.

To calculate the DP (degree of polymerization) of PBLG block, the total integral area of peaks *b, c* is defined as A_{bc} and set as 180.00, with which the integral area of peak *g*, defined as A_g, can be therefore obtained as 110.08. Then the DP of the PBLG block should satisfy the following equation:

$$n = \frac{A_g}{2} = \frac{110.08}{2} \approx 55$$

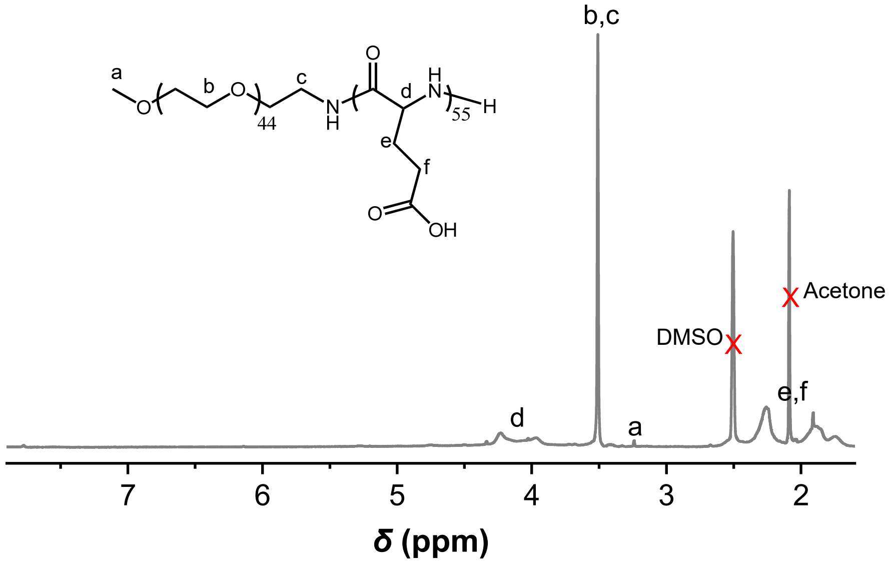

Figure S4. ^{1}H NMR spectrum of PEG_{45}-*b*-PGA_{55} in DMSO-d_6.

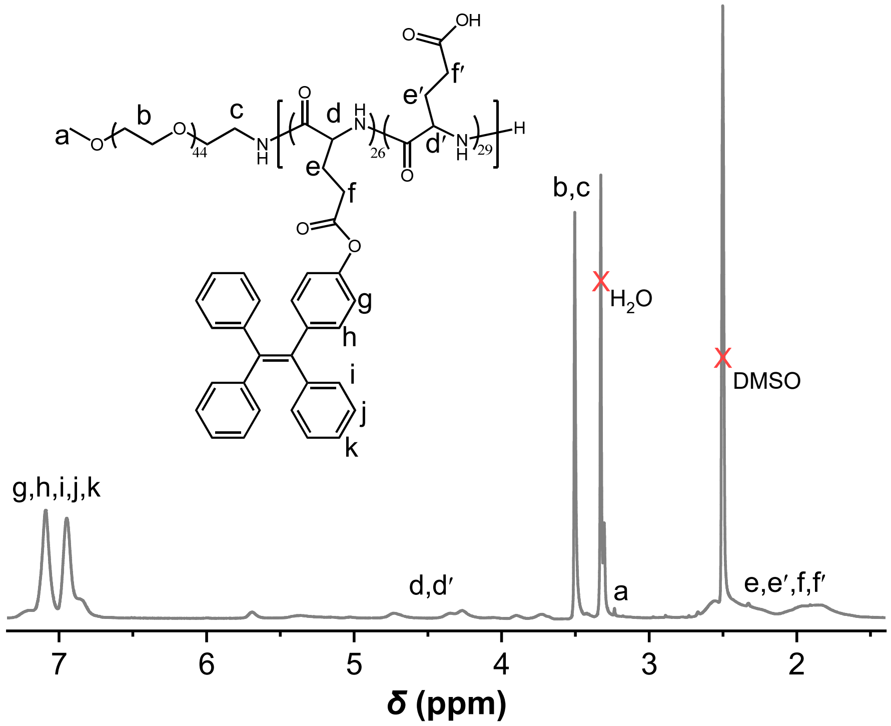

Figure S5. ^{1}H NMR spectrum of PEG_{45}-*b*-P($GATPE_{26}$-*stat*-GA_{29}) in DMSO-d_6.

To calculate the grafting number of TPE, the total integral area of peaks *b, c* is defined as A_{bc} and set as 180.00, with which the integral area of peak *j, k, l, m, n*, defined as A_{jklmn}, can be therefore obtained as 488.96. Then the grafting number of TPE should satisfy the following equation:

$$x = \frac{A_{jklmn}}{19} = \frac{488.96}{19} \approx 26$$

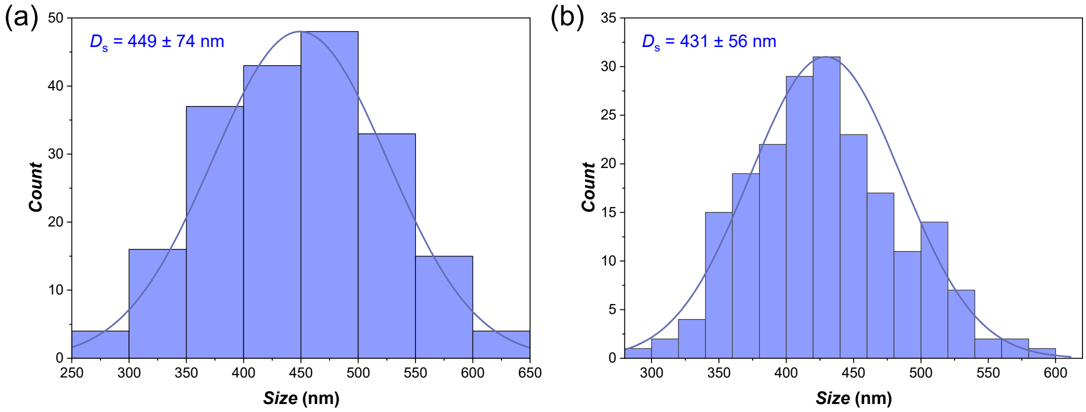

Figure S6. Size distribution of the nanocucurbits calculated from TEM images (a) or SEM images (b). D_s refers to the statistical diameter of nanocucurbits.

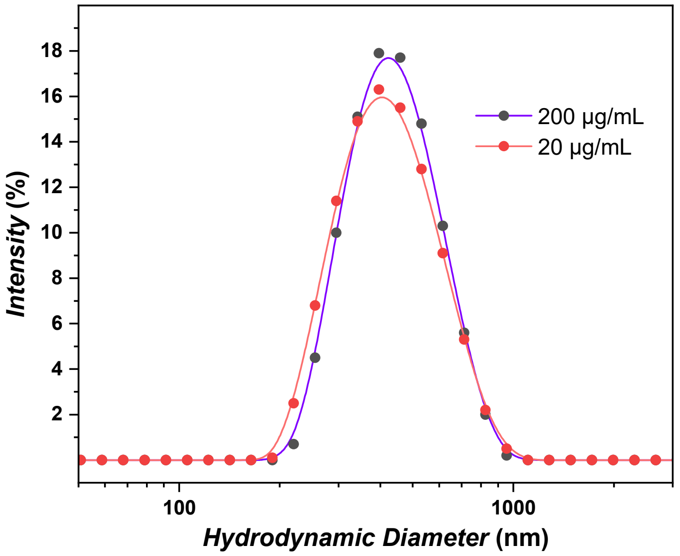

Figure S7. Dilution stability of the nanocucurbits aqueous solution.

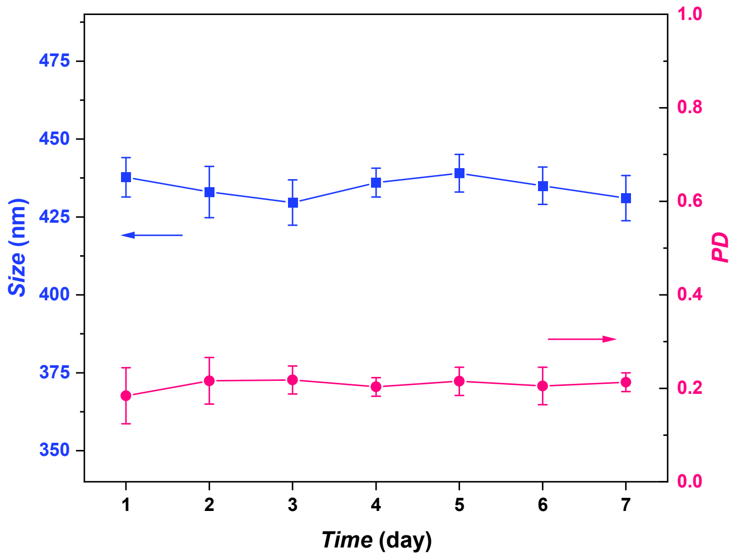

Figure S8. Hydrodynamic diameters and polydispersity of the nanocucurbits aqueous solution at different storage periods.

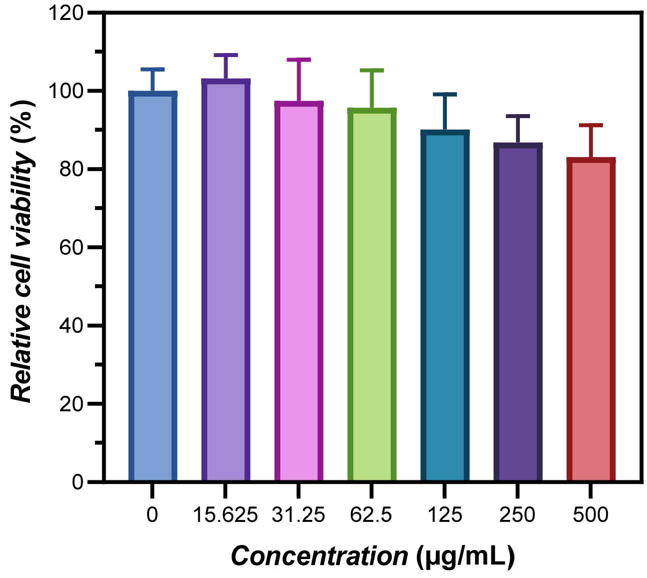

Figure S9. Relatively cell viabilities of the nanocucurbits against HepG2 cells at different concentrations.

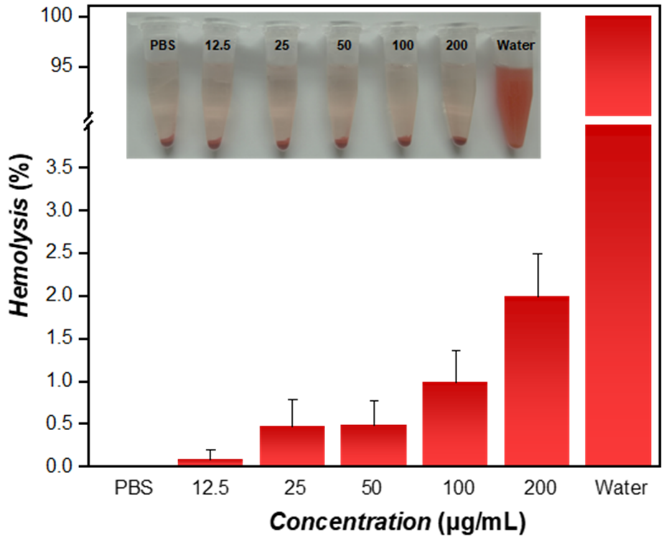

Figure S10. Hemolysis rate of the nanocucurbits.

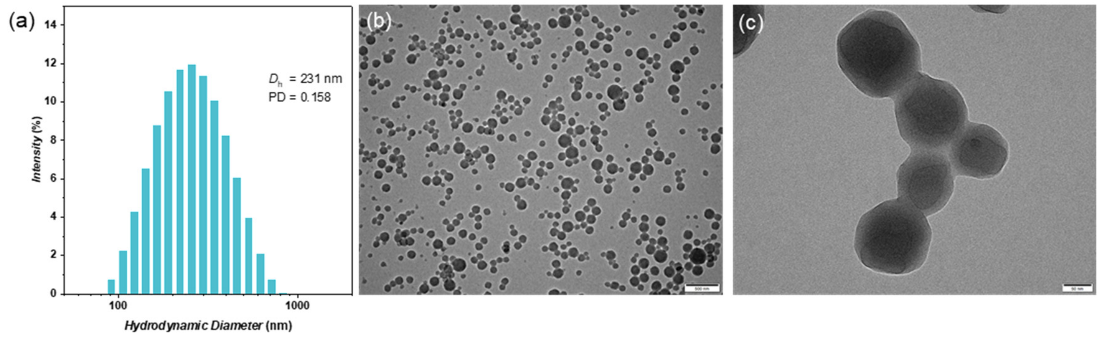

Figure S11. DLS studies (a) and TEM images (b, c) of the spherical micelles.

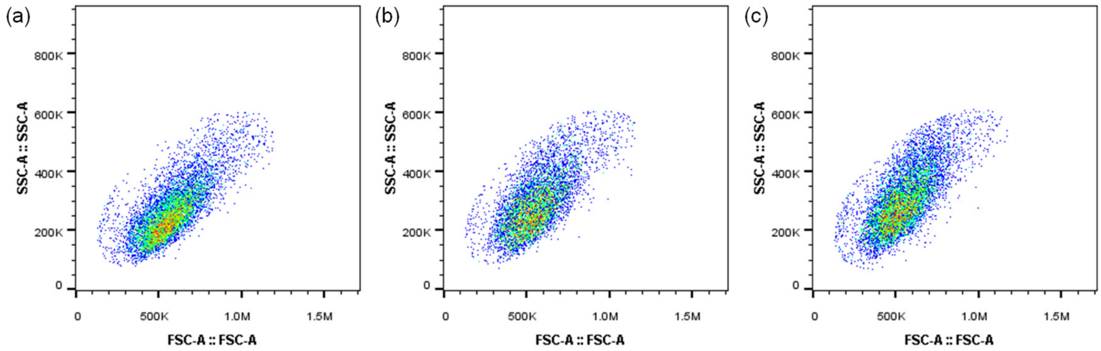

Figure S12. Flow cytometry analysis. Dot plots showing side scattering (SSC) and forward scatter (FSC) of HepG2 cells in control group (a), spherical micelles (b), and the nanocucurbits.

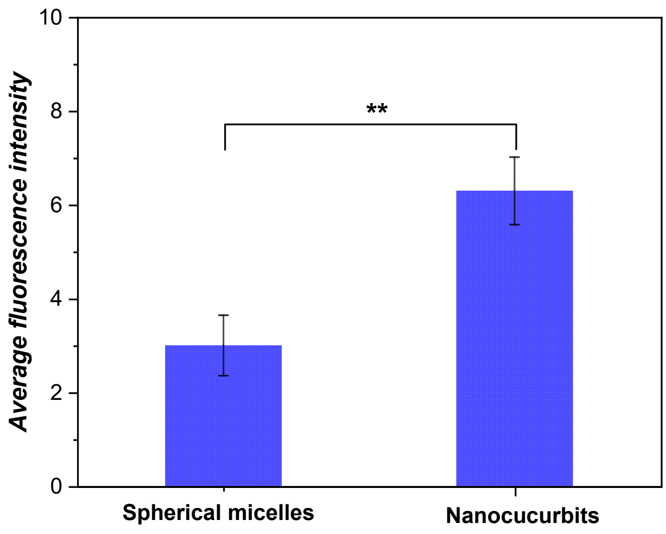

Figure S13. Statistical analysis of average blue fluorescence intensity from CLSM images (n = 3). p values were calculated by one-way ANOVA. *$p < 0.05$, **$p < 0.01$, ***$p < 0.001$.

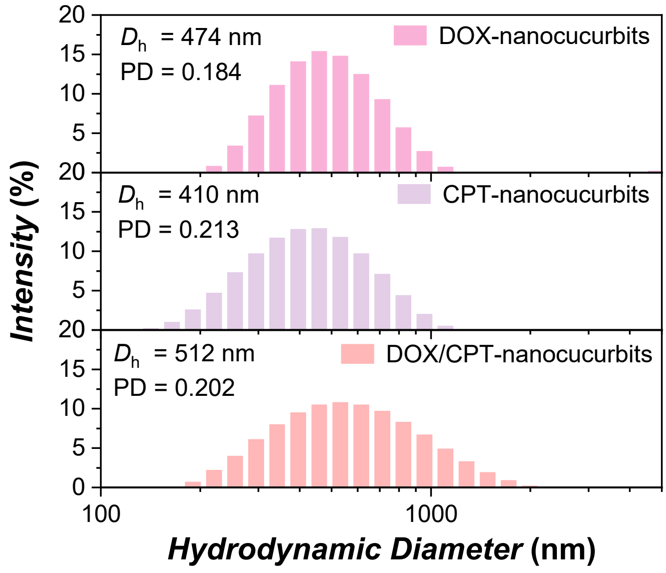

Figure S14. DLS results of DOX-nanocucurbits, CPT-nanocucurbits, and DOX/CPT-nanocucurbits.

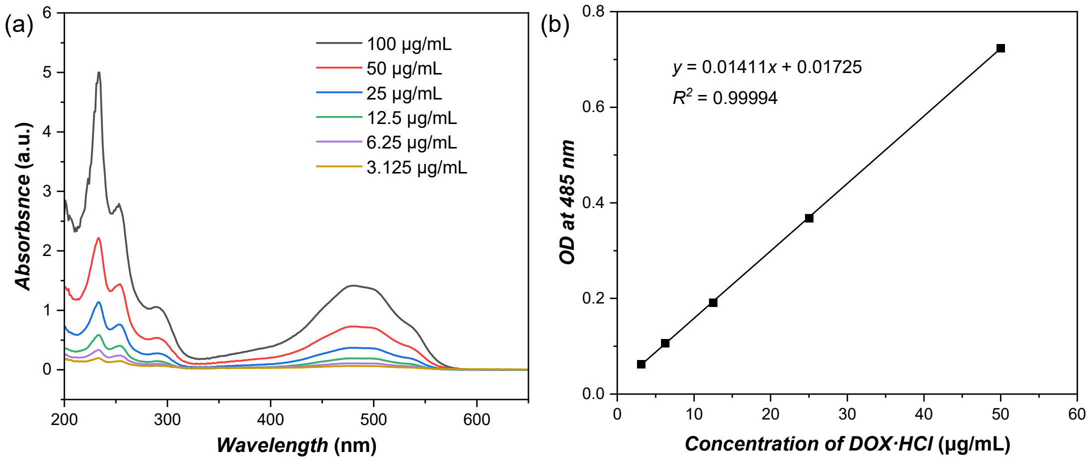

Figure S15. The standard curves of DOX·HCl measured by an ultraviolet-visible spectroscopy.

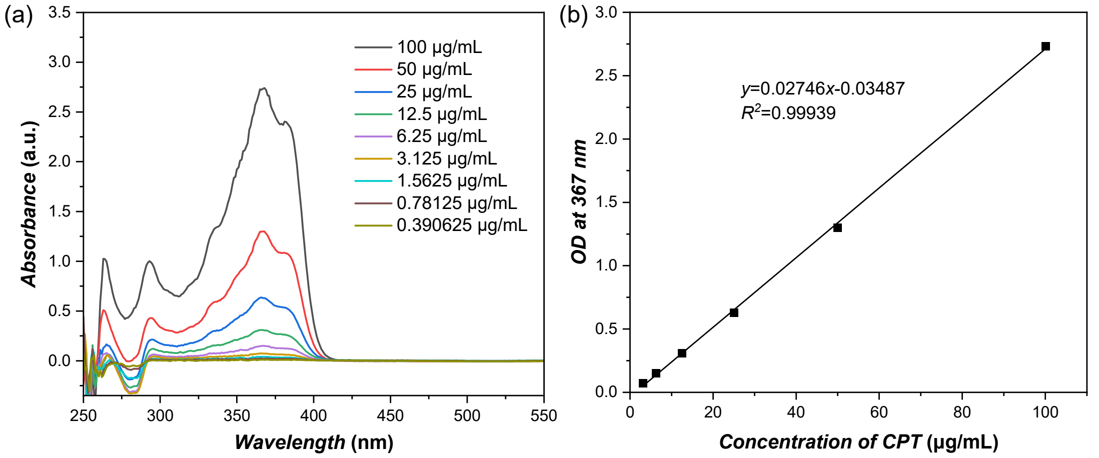

Figure S16. The standard curves of CPT in THF/DMF mixture (v/v = 4:1) solution measured by an ultraviolet-visible spectroscopy.

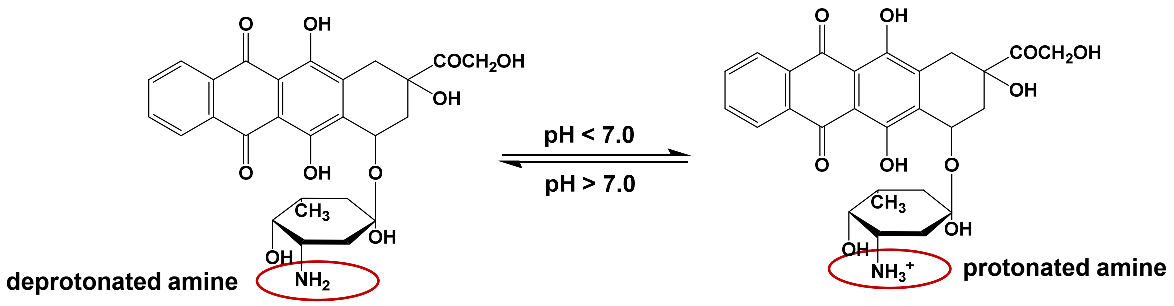

Figure S17. Ionization of doxorubicin in different pH media.

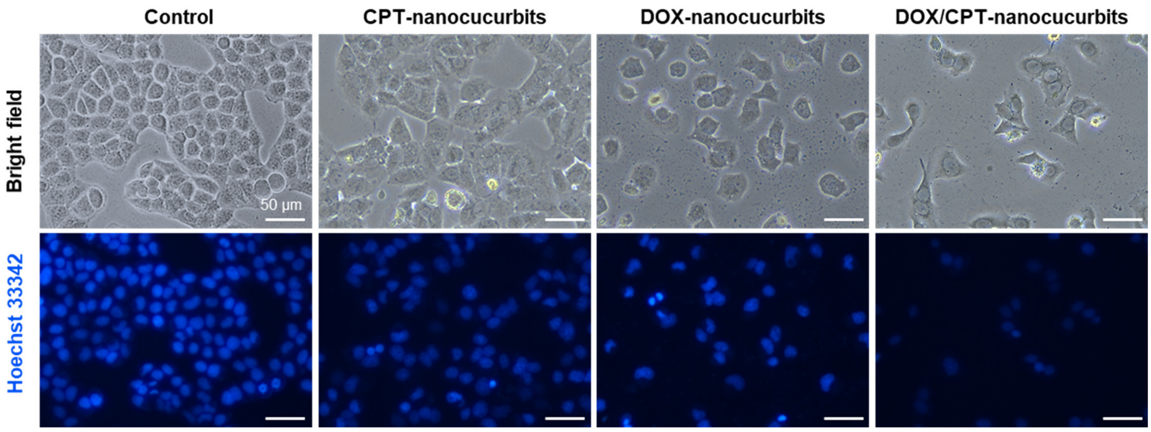

Figure S18. Representative fluorescence images of HepG2 cells stained with Hoechst 33342 after different treatments. Scale bar: 50 μm.

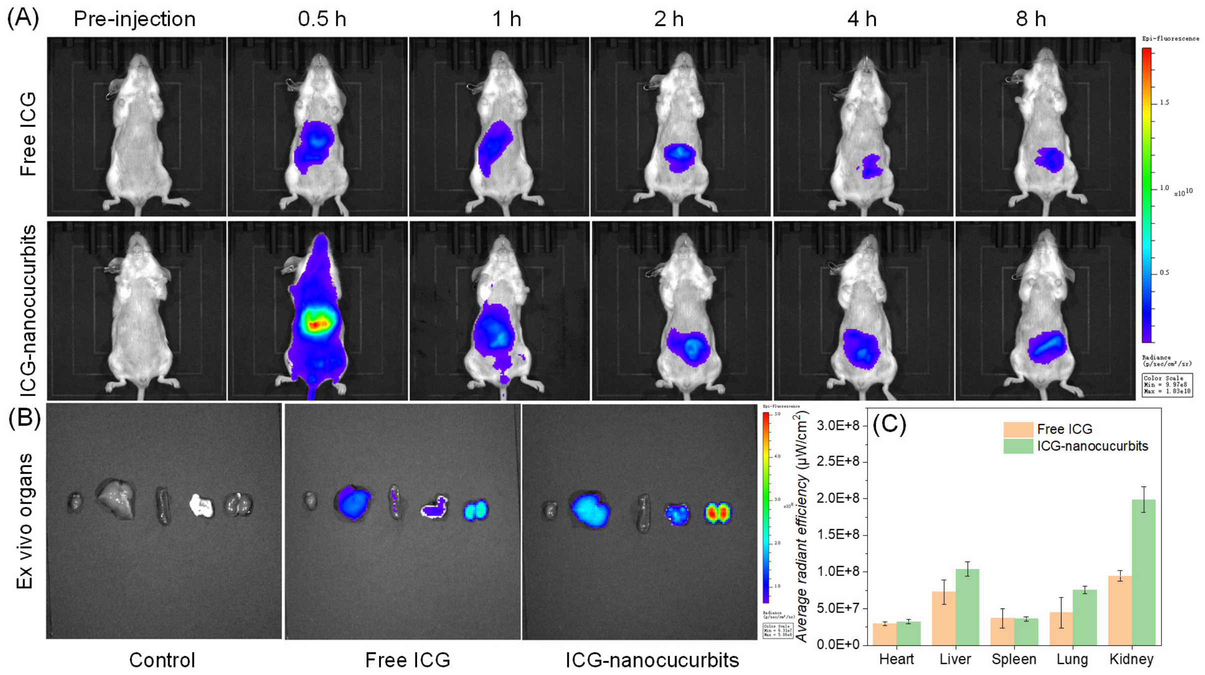

Figure S19. In vivo biodistribution. (A) In vivo fluorescence images of NOG mice at different time points post intravenous injection of free ICG and ICG-nanocucurbits, respectively. (B) Ex vivo fluorescence images of major organs (heart, liver, spleen, lung, and kidney) dissected from mice at 8 h post-injection. (C) Average radiant efficiency analysis of the main organs (n = 3). The untreated control group was utilized to minimize background interference.

Reference

[1] Wei, P.; Chen, S.; Shi, J.; Du, J., Oxygen-Generating Polymer Vesicles for Enhanced Sonodynamic Tumor Therapy. *J. Controlled Release* **2023,** *353*, 975.
